# Childhood exposures to environmental chemicals and neurodevelopmental outcomes in congenital heart disease

**DOI:** 10.1371/journal.pone.0277611

**Published:** 2022-11-17

**Authors:** J. William Gaynor, Nancy B. Burnham, Richard F. Ittenbach, Marsha Gerdes, Judy C. Bernbaum, Elaine Zackai, Daniel J. Licht, William W. Russell, Erin E. Zullo, Thomas Miller, Hakon Hakonarson, Kayan A. Clarke, Gail P. Jarvik, Antonia M. Calafat, Asa Bradman, David C. Bellinger, Frederick M. Henretig, Eric S. Coker

**Affiliations:** 1 Division of Cardiothoracic Surgery, Department of Surgery, Children’s Hospital of Philadelphia, and the Perelman School of Medicine, University of Pennsylvania, Philadelphia, PA, United States of America; 2 Division of Biostatistics and Epidemiology, Cincinnati Children’s Hospital Medical Center, Cincinnati, OH, United States of America; 3 Department of Psychology, Children’s Hospital of Philadelphia, and the Perelman School of Medicine, University of Pennsylvania, Philadelphia, PA, United States of America; 4 Department of Pediatrics, Children’s Hospital of Philadelphia, and the Perelman School of Medicine, University of Pennsylvania, Philadelphia, PA, United States of America; 5 Division of Genetics, Department of Pediatrics, Children’s Hospital of Philadelphia, and the Perelman School of Medicine, University of Pennsylvania, Philadelphia, PA, United States of America; 6 Division of Neurology, Department of Pediatrics, Children’s Hospital of Philadelphia, and the Perelman School of Medicine, University of Pennsylvania, Philadelphia, PA, United States of America; 7 Division of Pediatric Cardiology, Maine Medical Center, Portland, ME, United States of America; 8 The Center for Applied Genomics, The Children’s Hospital of Philadelphia and the Perelman School of Medicine, University of Pennsylvania, Philadelphia, PA, United States of America; 9 Department of Environmental and Global Health, University of Florida, Gainesville, FL, United States of America; 10 Departments of Medicine (Division of Medical Genetics) and Genome Sciences, University of Washington Medical Center, Seattle, WA, United States of America; 11 Division of Laboratory Sciences, National Center for Environmental Health, Atlanta, GA, United States of America; 12 Department of Public Health, University of California, Merced, Merced, CA, United States of America; 13 Department of Neurology, Boston Children’s Hospital and Harvard Medical School, Boston, MA and Department of Environmental Health, Harvard T.H. Chan School of Public Health, Boston, MA, United States of America; 14 Emergency Medicine, Children’s Hospital of Philadelphia and the Perelman School of Medicine, University of Pennsylvania, Philadelphia, PA, United States of America; King Faisal Specialist Hospital and Research Center, SAUDI ARABIA

## Abstract

**Background:**

Children with congenital heart defects have an increased risk of neurodevelopmental disability. The impact of environmental chemical exposures during daily life on neurodevelopmental outcomes in toddlers with congenital heart defects is unknown.

**Methods:**

This prospective study investigated the impacts of early childhood exposure to mixtures of environmental chemicals on neurodevelopmental outcomes after cardiac surgery. Outcomes were assessed at 18 months of age using The Bayley Scales of Infant and Toddler Development-III. Urinary concentrations of exposure biomarkers of pesticides, phenols, parabens, and phthalates, and blood levels of lead, mercury, and nicotine were measured at the same time point. Bayesian profile regression and weighted quantile sum regression were utilized to assess associations between mixtures of biomarkers and neurodevelopmental scores.

**Results:**

One-hundred and forty infants were enrolled, and 110 (79%) returned at 18 months of age. Six biomarker exposure clusters were identified from the Bayesian profile regression analysis; and the pattern was driven by 15 of the 30 biomarkers, most notably 13 phthalate biomarkers. Children in the highest exposure cluster had significantly lower adjusted language scores by -9.41 points (95%CI: -17.2, -1.7) and adjusted motor scores by -4.9 points (-9.5, -0.4) compared to the lowest exposure. Weighted quantile sum regression modeling for the overall exposure-response relationship showed a significantly lower adjusted motor score (β = -2.8 points [2.5^th^ and 97.5^th^ percentile: -6.0, -0.6]). The weighted quantile sum regression index weights for several phthalates, one paraben, and one phenol suggest their relevance for poorer neurodevelopmental outcomes.

**Conclusions:**

Like other children, infants with congenital heart defects are exposed to complex mixtures of environmental chemicals in daily life. Higher exposure biomarker concentrations were associated with significantly worse performance for language and motor skills in this population.

## Introduction

Improved survival after surgical repair of congenital heart defects (CHD) has led to recognition that neurobehavioral disability, including mild cognitive dysfunction, impaired motor skills, language difficulties, and impaired attention and executive function, is the most common and potentially most disabling long-term adverse outcome of infants with CHD [1]. Despite the efforts of multiple investigators and clinicians, there has been minimal progress in improving early neurodevelopmental outcomes for these children over the past 20 years [2]. For many years, studies of neurodevelopmental outcomes for children with CHD were focused on cardiac surgery; specifically on intraoperative management strategies [2]. This focus was understandable inasmuch as these strategies are potentially modifiable and thus there were potential opportunities to improve outcomes. However, recent studies have shown that innate patient factors (prematurity, brain dysmaturity, genetic anomalies, maternal education, etc.) eclipse intraoperative factors as predictors of worse neurodevelopmental outcomes [1, 2]. Moreover, multiple studies have demonstrated that currently recognized risk factors explain only a small portion of total variation in neurodevelopmental outcomes [2]. A better understanding of other modifiable, yet under recognized factors, is critical in not only identifying risk profiles, but also in guiding development of more individualized therapeutic strategies.

There is growing evidence of the adverse effects of even low level, early life exposures to environmental chemicals on neurodevelopment (e.g., metals, tobacco smoke, pesticides, industrial chemicals [phthalates, phenols, parabens]) [3, 4]. Many of these chemicals are common in the environment and are known to be endocrine disrupting compounds (EDCs) [3, 5–10]. Household dust can contain lead (Pb), phthalates, and pesticides [11, 12]. Phthalates, phenols, and parabens are present in many common consumer and personal care products [13]. In some cases (e.g. Pb), there is no known “safe” threshold for exposure to these environmental chemicals, with infants having increased exposure vulnerability and biologic susceptibility compared to other sub-populations. The early postnatal period through the first two years of life is a particularly important time for brain development [1, 4]. The infant brain is in a state of rapid growth; thus, impairment of brain cognitive function may arise from relatively low levels of exposure [3, 4]. Many previous epidemiologic studies have focused on exposure to one neurotoxicant at a time [4]. However, environmental chemical exposures occur as complex mixtures of chemicals from multiple chemical classes [4]. Several recent studies have suggested that exposure to environmental chemical mixtures may have cumulative or possibly synergistic (or antagonistic) adverse effects on neurodevelopment [4, 14–17].

We have previously shown that, in the perioperative period, neonates undergoing cardiac surgery with cardiopulmonary bypass (CPB) are exposed to high-levels of phthalates and phenols from medical devices [18]. Cyclohexanone is a major industrial solvent used in the production of medical devices [19]. Everett and colleagues found substantial exposure to cyclohexanone in neonates undergoing CPB [19]. Higher exposures were associated with worse performance for cognitive and language skills at 12 months of age. However, to our knowledge, no previous study has investigated the impact of exposure to environmental chemicals (in isolation or as part of a mixture) encountered during daily life on neurodevelopmental outcomes in the CHD population. It is important to realize that in the infant CHD population, these chemical exposures co-occur with other neurodevelopmental risks (e.g., brain dysmaturity, hypoxia, cardiac surgery, etc.), which may exacerbate the risk of neurotoxicity [1, 4]. In this context, children with CHD are a particularly at-risk and vulnerable population.

The purpose of this study is to determine if early life chemical exposures are potentially important factors which are not currently included in our understanding of the mechanisms underlying neurodevelopmental disability in children with CHD. We assessed a large number (*n* = 30) of exposure biomarkers of neurotoxicants and EDCs, including Pb, mercury, environmental tobacco smoke (cotinine, nicotine), pesticides, phthalates, phenols, and parabens.

### Patients and methods

We conducted a prospective, observational cohort study investigating the effect of childhood exposure to mixtures of environmental chemicals on neurodevelopmental outcomes at 18 months of age after cardiac operations in newborns. Inclusion criteria were: (1) infants with CHD and an expected operation with cardiopulmonary bypass (CPB) at age 44 weeks post-conception or younger. Exclusion criteria were (1) presence of an identified genetic syndrome, (2) major extracardiac anomaly, or (3) language other than English spoken in the home. The Children’s Hospital of Philadelphia (CHOP) Institutional Review Board and the University of Florida Institutional Review Board approved the study. Written informed consent was obtained from the parent or guardian. The involvement of the Centers for Disease Control and Prevention (CDC) laboratory did not constitute engagement in human subjects research.

Patients were enrolled between September 1, 2011 and August 31, 2015. Operations were performed by 1 of 4 cardiac surgeons with a dedicated team of cardiac anesthesiologists. Deep hypothermic circulatory arrest (DHCA) was used at the surgeon’s discretion. Modified ultrafiltration was performed in all patients. Patient-related variables (e.g., age at testing, sex, race, anthropometric measures) and peri-operative variables (e.g., age at surgery, bypass support times, hematocrit, length of stay) were collected from patient records. Newborn length, weight, and head circumference were measured, and the z-score for each growth measurement was calculated using World Health Organization (WHO) standards for full-term infants and the Fenton growth chart for preterm infants [20, 21]. Body mass index (BMI) and z-scores were calculated using WHO standards for full-term infants and the Olsen growth chart for preterm infants [22]. Maternal education, socioeconomic-related variables that comprise the Hollingshead socioeconomic status (SES), and ethnicity were determined through parental report [23]. A blood sample was obtained from each patient for genetic analysis. Whole exome sequencing was performed on subjects and all consented parents. Details are included in the Supplemental Files. Variant calls were queried for single nucleotide polymorphisms to determine apo-lipoprotein E (*APOE)* genotype according to the following classification of bases at rs429358 and rs7412, respectively on chromosome 19: C,T, ε1; T,T, ε2; T,C, ε3; C,C, ε4.

A study follow-up visit was conducted at 18 (mean) ± 1 (SD) months of age. Growth parameters (weight, height, and head circumference, BMI) were measured, and z-scores derived using WHO standards. Collection of spot infant urine samples was performed by placing a cotton ball in the diaper. The cotton balls were placed into a 3 to 5 mL polypropylene syringe (Becton-Dickinson, Franklin Lakes, NJ), and the urine was transferred into a polypropylene Cryovial (Simport, Beloeil, QC, Canada). Specific gravity (SG) was measured at room temperature using a handheld refractometer. Urine and negative control samples were frozen at –20°C and shipped overnight on dry ice to the CDC. At the CDC, urinary biomarkers were quantified in infant urine using solid phase extraction coupled to high performance liquid chromatography-isotope dilution tandem mass spectrometry after enzymatic deconjugation to hydrolyze urinary conjugates and following standard quality assurance/quality control procedures as described in detail previously [24–27]. The target chemical biomarkers measured and their acronyms are shown in Table 1. The limits of detection (LOD) were 0.1–1.7 μg/L, depending on the biomarker. A venous blood sample was obtained for measurement of lead, mercury, nicotine, and cotinine. Lead levels for Pennsylvania residents were performed by the CHOP Clinical Laboratory. Measurements for out of state subjects were performed by ARUP Laboratories (Salt Lake City, UT). Total mercury measurements for all subjects were performed by ARUP Laboratories. Nicotine and cotinine were measured by ARUP Laboratories and NMS Labs (Horsham, PA).

**Table 1:** Exposure biomarkers and chemical class.

**Herbicides**
2,4-D (2,4-Dichlorophenoxyacetic acid)
**Organophosphates**
TCPY (3,5,6-Trichloro-2-pyridinol)
**Pyrethroids**
3-PBA (3-Phenoxybenzoic acid)
**DEET**
DCBA (3-(Diethylcarbamoyl) benzoic acid)
**Phenols, Parabens, Trichlocarbans**
2,4-DCP (2,4-Dichlorophenol)
2,5-DCP (2,5-Dichlorophenol)
BP-3 (Benzophenone-3)
BPA (Bisphenol A)
BPB (Butyl Paraben)
BPS (Bisphenol S)
PPB (Propyl Paraben)
TCC (Triclocarban)
TCS (Triclosan)
**Phthalates**
MBP (Mono-n-butyl phthalate)
MBzP (Monobenzyl phthalate)
MCNP (Mono carboxyisononyl phthalate)
MCOP (Mono carboxyisooctyl phthalate)
MCPP (Mono-3-carboxypropyl phthalate)
MECPP (Mono-2-ethyl-5-carboxypentyl phthalate)
MEHHP (Mono-2-ethyl-5-hydroxyhexyl phthalate)
MEHP (Mono-2-ethylhexyl phthalate)
MEOHP (Mono-2-ethyl-5-oxohexyl phthalate)
MEP (Monoethyl phthalate)
MHBP (Mono-hydroxybutyl phthalate)
MHiBP (Mono-hydroxyisobutyl phthalate)
MIBP (Mono-isobutyl phthalate)
MNP (Mono-isononyl phthalate)
**Phthalate alternatives (DINCH)**
MCOCH (cyclohexane-1 2-dicarboxylic acid monocarboxyisooctyl ester)
MHINCH (Cyclohexane-1 2-dicarboxylic acid monohydroxy isononyl ester)
**Other Exposures**
Lead
Mercury
Nicotine
Cotinine

Patients were evaluated by a genetic dysmorphologist. Neonatal recognition of dysmorphic features may be difficult; therefore, some patients for whom the diagnosis of a genetic syndrome was made at a later evaluation were enrolled. Genetic testing was performed as clinically indicated. Results of the genetic evaluations were classified as normal if no genetic or chromosome abnormality was demonstrated, abnormal if a specific diagnosis was confirmed, and suspect if there was evidence of a genetic syndrome that could not be confirmed. Neurodevelopmental outcomes were assessed using The Bayley Scales of Infant and Toddler Development-III; which provide composite scores for cognitive, language and motor skills (μ = 100 with σ = 15) [28]. Higher scores indicate better skills.

### Statistical analysis

Data analysis proceeded in three phases: a descriptive analysis phase to fully characterize the study population; a modeling phase in which selected neurocognitive measures were regressed onto a series of established risk factors to identify influential covariates for subsequent modeling; and, finally, a health effects modeling approach which explicitly evaluated the effects of early-childhood chemical mixtures on neurodevelopmental attainment. All data were analyzed in SAS (v9.4) or R (v 4.1.2).

### Descriptive statistics

Measures of central tendency, variability, and univariate associations with neurodevelopmental outcomes were computed for patient-related, peri-operative, and post-operative variables and environmental exposure biomarkers. Of the analytes used in this study, several returned nondetectable concentrations for some participants and required imputation consistent with established methods in the literature [29, 30]. As such, LOD/sqrt(2) was used to impute values for the two analytes for which the rate of non-detection was low (< 20%) while single imputation was used for the three analytes for which non-detection was more moderate (20 to 50%). For single imputation, specifically, values were drawn from a randomly generated lognormal probability distribution with imputed values bounded between zero and the analyte’s LOD. A total of 453 of 3190 (14.2%) biomarker concentrations were reported as <LOD, and 121 of 3190 (3.8%) concentrations were reported as missing because of insufficient urine volume for analysis or analytical interferences. If the CDC laboratory reported numeric values for concentrations <LOD, these values were used as observed concentrations. To help characterize relationships among the 30 analytes reported here, a table of Spearman-rho correlation coefficients was generated. Because of the extremely large number of bivariate correlations (*n* = 870) in the matrix, only those achieving statistical significance at an adjusted α = 0.0024 level for 30 moderately correlated endpoints are presented in S2 Table. All observed and imputed urine concentrations were corrected for specific gravity using Levine’s formula [31].

$$Result_{ADJ}\;(ng/mL)=Result\times [(SG_{Median}-1.000)/(SG_{Testing}-1.000)]$$

Where Result = observed concentration of an analyte at testing

Result_ADJ_ = observed concentration of an analyte at testing after adjusting for specific gravity.

SG_Median_ = median specific gravity value across all subjects.

SG_Testing_ = concentration of a subject’s urine at the time of testing.

### Neurocognitive modeling

Cognitive, Language, and Motor Composite scores from the Bayley Scales of Infant and Toddler Development obtained at 18 months of age were regressed onto a series of 29 patient-related, peri-operative, and post-operative covariates, individually, using a generalized linear model (normal distribution, identity link) specifically to identify relevant covariates for subsequent modeling of chemical mixtures effects on neurodevelopment. The covariates selected for use in this study were deemed relevant for modeling based upon our prior work, as well as the published literature in CHD [2, 32, 33]. We elected to keep as many potentially influential covariates as possible for subsequent, in-depth analysis. Assumptions and distributional properties were tested and deemed amenable for analysis. Two of the outcomes were skewed, requiring a Box-Cox transformation to achieve normality and make them amenable for parametric analysis (Cognitive Composite Score^^2.25^, Motor Composite Score^^2.75^). Single covariate models with Wald-statistic *p*-values < 0.20 were used as candidates for inclusion in a best-fitting multiple covariate model for each outcome. Final, best-fitting, multiple-covariate models were then selected based on individual and model-specific likelihood ratio tests, AIC, and BIC values. Covariates used in the final, best-fitting models were then used in subsequent modeling that assessed the neurodevelopmental health effects of the environmental chemical mixture (described in turn).

### Modeling the effects of environmental chemical mixtures on neurodevelopment

Because the purpose of this study is to determine if early life chemical exposures are potentially important contributors to our understanding of the mechanisms underlying neurodevelopmental disability in children with CHD, two additional semi-parametric modeling techniques were used to investigate the impact of exposures to environmental chemicals.

### Bayesian profile regression (BPR)

BPR is a semi-parametric clustering algorithm that is set in a Bayesian framework with Markov Chain Monte Carlo (MCMC) sampling [34, 35]. In this study, BPR was used to identify environmental exposure patterns among the children that could be linked to our study outcomes using conventional linear regression [36]. Advantages of using BPR compared to more conventional clustering algorithms such as K means, Euclidean distance, or nearest neighbor clustering, include a lack of constraint on defining the number of clusters a priori, allowances for categorical covariates on which to base the clusters, its ability to handle and account for correlated, as well as missing data, and a variable selection option to help identify the covariates most responsible for observed patterns of clusters.

Missing exposure (or covariate data) values are accommodated with BPR by denoting each missing value as ‘NA’ and applying multiple imputation throughout the sampler using the full model information [35, 37]. Another particular advantage of BPR is the ability to handle categorical data in the clustering. Hence, if data are highly skewed, they can be reclassified to accommodate the clustering algorithm. In our case, highly skewed data were reclassified into quantiles then fit as categorical covariates, such that those with similarly high levels of exposure (i.e., highest quantile) are clustered together and those with similarly low levels of exposure (i.e., lowest quantile) are clustered together” [34, 35].

As mentioned, prior to fitting the model, we first transformed urinary biomarker concentrations into tertiles based on the sample size of our cohort and the highly skewed nature of the biomarker data. Target exposure biomarkers were fit as categorical (discrete) covariates and the variable selection option was utilized to identify exposure biomarkers of interest driving the observed clustering patterns. The PReMiuM package (version 3.2.6) available in the R Statistical Computing Platform (version 4.1.2) was used to implement BPR [34]. Default priors available in the PReMiuM package were used. The outcome was excluded from the cluster analysis so that cluster membership is not influenced by neurodevelopmental outcomes but rather only the co-exposure patterns. In the second step, neurodevelopmental outcomes were analyzed using linear regression models. These outcomes included Cognitive Composite score, Motor Composite score, and Language Composite score. The clusters were fit as categorical variables for adjusted as well as unadjusted linear regression models. In each outcome-specific model, the cluster with the lowest exposure biomarker concentrations was set as a reference group. Clusters with regression coefficient estimates that had 95% confidence intervals that did not overlap with zero were considered significantly associated with the neurodevelopmental outcome of interest.

### Weighted quantile sum regression (WQS)

WQS is an alternative statistical method designed to assess associations between correlated joint exposures with a health outcome of interest. With WQS, exposure biomarker concentrations are transformed into quantiles and joined into a weighted index, which both reduces the model parameter dimensions and addresses multi-collinearity. An overall unidirectional effect of the mixture is estimated, with individual exposure biomarkers ranked (or weighted) based on their relative contribution to the weighted quantile index. In our implementation of WQS, we performed 100 bootstrap samples for parameter estimation along with 100 repeated holdouts with cross-validation. Model training and validation were performed using a 40%/60% split of the cohort data. We applied the repeated holdouts because it has recently been shown to improve the stability of WQS estimates in the context of small cohort sample sizes [38]. Since WQS handles missing values by automatically imputing to the lowest exposure group, which would be an inappropriate assumption, only 26 chemical biomarkers were included in the WQS model fitting. We fit WQS using tertiles for ranking of each exposure biomarker and assumed a negative exposure-response for the WQS index since we are interested in adverse effects of the chemical biomarkers mixture on neurodevelopment indicators. A sensitivity analysis was performed which allowed for a positive exposure-response for the exposure mixture; however, there was no evidence of a significant positive effects (data not shown). The health outcome-specific models were modeled as a linear function of the WQS index and covariate adjustment was performed using the same covariates for the linear regression analysis as described above. In our presentation of results, our inference was based on the overall mean WQS index effect estimate along with the corresponding 95% confidence intervals. We evaluated the importance of the estimated weights for each exposure biomarker using a threshold of ≥ 0.038 (1/26 [number of exposure biomarkers]) as this is shown to be a good indicator of variable importance [38, 39].

## Results

**Fig 1:**
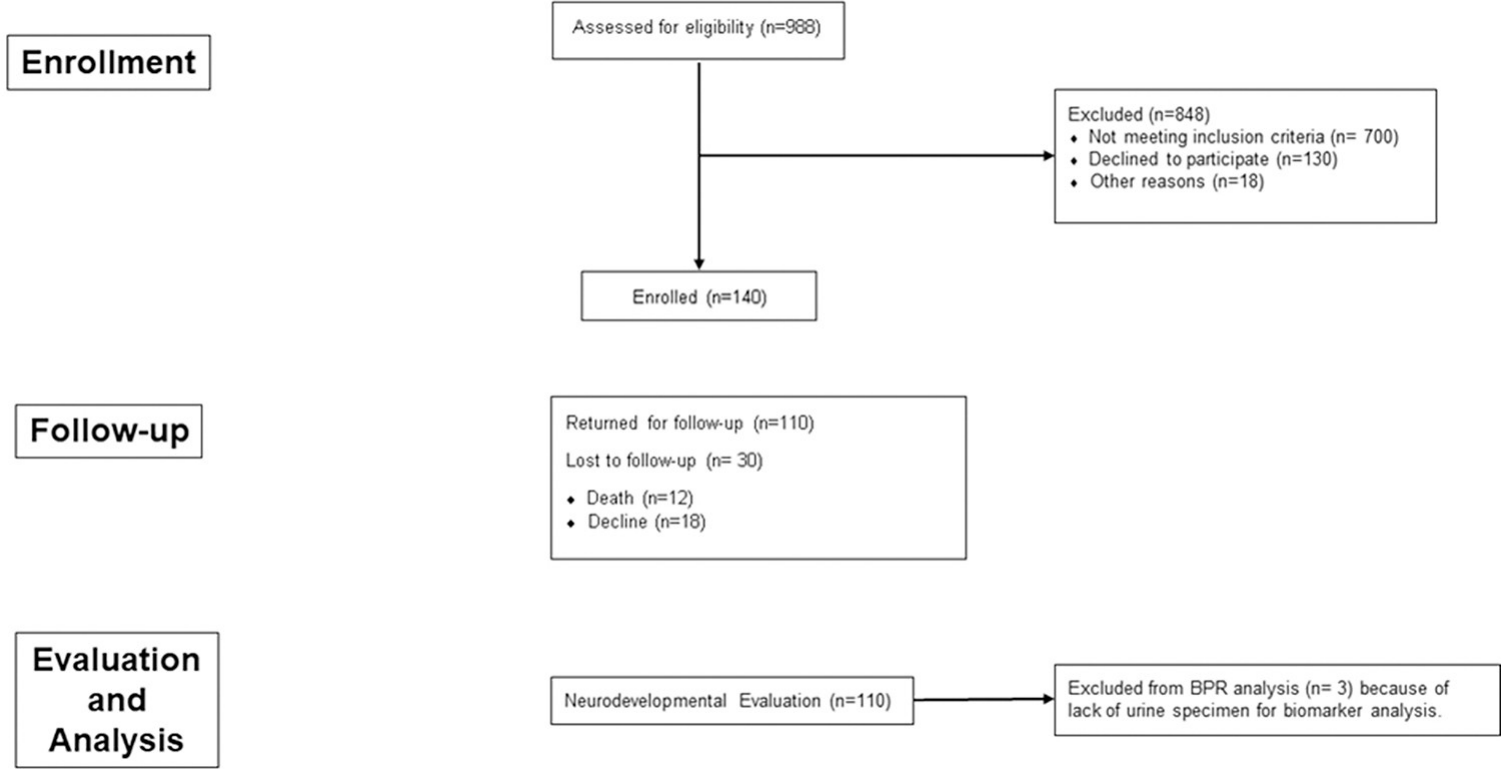
Study enrollment and evaluation flowchart.

Between September 1, 2011 and August 31, 2015, 140 infants were enrolled in the study. Of these, 110 (79%) returned for the evaluation at 18 months of age (Fig 1). The types of CHD included hypoplastic left heart syndrome or variant (*n* = 32), transposition of the great arteries (*n* = 39), and other (*n* = 39). Patent characteristics and operative management variables are shown in Table 2. The only significant differences were larger placenta weight/birth weight and longer DHCA time in the non-returners.

**Table 2:** Descriptive statistics and comparisons for returners (n = 110) vs. non-returners (N = 30).

Variable	Returners		Returners		Returners	Non-returners	*p*-value
	*n*	Mdn (IQR)	*n*	Mdn (IQR)	*f* (%)	*f* (%)	
*Maternal Characteristics*							
Placental Weight/Birth Weight	89	0.1 (0.1, 0.2)	16	0.2 (0.1, 0.2)			**< 0.01**
Maternal Age at Childbirth	110	31.5 (27.0, 35.0)	20	31.5 (25.5, 34.0)			1.00
Hollingshead Raw Score	109	50.0 (38.0, 55.5)	16	48.8 (25.0, 57.0)			0.61
Gestational Diabetes							
No					106 (96.4)	20 (100.0)	1.00
Yes					4 (3.6)	0 (0.0)	
Gestational Hypertension							
No					109 (99.1)	19 (95.0)	0.28
Yes					1 (0.9)	1 (5.0)	
Preeclampsia							
No					107 (97.3)	18 (90.0)	0.17
Yes					3 (2.7)	2 (10.0)	
Smoke Exposure							
No					100 (90.9)	17 (89.5)	0.69
Yes					10 (9.1)	2 (10.5)	
Multiple Births							
No					103 (93.6)	19 (95.0)	1.00
Yes					7 (6.4)	1 (5.0)	
Impaired Maternal-Fetal Environment							
No					92 (83.6)	16 (80.0)	0.75
Yes					18 (16.4)	4 (20.0)	
Maternal Education							
Partial High School					6 (5.4)	1 (6.2)	0.48
High School Graduate					10 (9.1)	2 (12.5)	
Partial College/Trade School					16 (14.6)	3 (18.8)	
College Diploma					50 (45.4)	4 (25.0)	
Graduate School					28 (25.4)	6 (37.5)	
*Patient Characteristics*							
Gestational Age	110	39.1 (38.4, 39.6)	20	39.1 (37.6, 39.3)			1.00
Birth Head Circumference (cm)	110	34.0 (33.0, 35.0)	20	33.0 (32.2, 34.2)			0.10
Birth Head Circumference *z*-score	110	-0.3 (-1.2), 0.8)	20	-0.7 (-1.6, 0.1)			0.26
Birth Length (cm)	110	49.5 (47.0, 51.5)	20	48.0 (47.0, 51.0)			0.65
Birth Length *z*-score	110	-0.1 (-1.0, 1.0)	20	-0.6 (-1.2, 0.8)			0.50
Birth Weight (kg)	110	3.3 (3.0, 3.6)	20	3.2 (2.8, 3.5)			1.00
Birth Weight *z*-score	110	0.0 (-0.7, 0.6)	20	-0.1(-1.1, 0.5)			1.00
Small for Gestational Age							
No					97 (88.2)	17 (85.0)	0.71
Yes					13 (11.8)	3 (15.0)	
Sex							
Female					42 (38.2)	11 (55.0)	0.22
Male					68 (61.8)	9 (45.0)	
Race							
Caucasian					89 (80.9)	12 (60.0)	0.08
African American					6 (5.4)	3 (15.0)	
Other					15 (13.6)	5 (25.0)	
Ethnicity							
Non-Hispanic					94 (85.4)	17 (89.5)	1.00
Hispanic					16 (14.5)	2 (10.5)	
Genetic Anomaly							
Normal					78 (70.9)	16 (80.0)	0.12
Confirmed					18 (16.40)	0 (0.0)	
Suspected					14 (12.7)	4 (20.0)	
APOE Genotype							
e2					8 (7.5)	3 (17.6)	0.35
e3					77 (72.0)	12 (70.6)	
e4					22 (20.6)	2 (11.8)	
Diagnosis Code							
HLHS					32 (29.1)	5 (25.0)	0.95
Transposition of the Great Arteries					39 (35.4)	8 (40.0)	
Other					39 (35.4)	7 (35.0)	
Preterm Birth							
No					101 (91.8)	19 (95.0)	1.00
Yes					9 (8.2)	1 (5.0)	
*Operative Management*							
Age at 1^st^ Operation (yrs.)	110	4.9 (3.0, 6.0)	20	3.5 (2.5, 10.5)			0.50
Weight at 1^st^ Operation (kg)	110	3.3 (3.0, 3.5)	20	3.4 (2.9, 3.7)			0.82
Total Support Time, 1^st^ Operation (mins)	110	74.5 (60.0, 98.0)	20	70.0 (54.0, 89)			0.50
Deep Hypothermic Circulatory Arrest Time, 1^st^ Operation (mins)	110	0.0 (0.0, 37.0)	20	8.0 (0.0, 37.0)			**< 0.01**
Class							
2 Ventricles, No Obstruction					53 (48.2)	11 (55.0)	0.98
2 Ventricles, Obstruction					17 (15.4)	3 (15.0)	
1 Ventricle, No Obstruction					8 (7.3)	1 (5.0)	
1 Ventricle, Obstruction					32(29.1)	5 (25.0)	
ECMO							
No					107 (97.3)	19 (95.0)	0.49
Yes					3 (2.7)	1 (5.0)	
*After First Operation*							
Length of Stay at 1^st^ Operation (days)	110	15.0 (11.0, 23.0)	20	13.5 (9.5, 21.0)			0.33
Number of Additional Operations with CPB							
None					66 (60.0)	12 (60)	1.00
1 or More					44 (40.0)	8 (40.0)	

*Notes*. IQR = interquartile range (25%^ile^, 75%^ile^). One-sample sign tests were used for continuous variables; Fisher Exact tests were used for categorical variables.

The mean Cognitive Composite score for the entire cohort (*n* = 110) was 93.2 ± 12.7. Cognitive Composite scores were more than 1SD below the expected mean for 19 subjects (17%) and more than 2SD below the expected mean for 3 (3%). The mean Language Composite score for the entire cohort was 91.9 ± 18.1. Language Composite scores were more than 1SD below the expected mean for 35 subjects (32%), and more than 2SD below the expected mean for 12 (12%). The mean Motor Composite score for the entire cohort was 92.0 ± 11.7. Motor Composite scores were more than 1SD below the expected mean for 24 subjects (22%), and more than 2SD below the expected mean for 5 (5%). With respect to the multiple covariate models for the Cognitive, Language, and Motor Composite scores, patient factors were more important predictors than operative management variables. (Table 3) Presence of a suspected or confirmed genetic anomaly was an independent predictor of worse performance on all three scores. Older gestational age was a predictor of a higher Cognitive score. Higher SES was associated with better Cognitive and Language Composite scores. Female sex was predictive of better performance for Language and Motor skills. Hispanic ethnicity was associated with worse Motor performance. Among the operative management factors, longer hospital length of stay for the initial hospitalization was associated with worse performance for the Motor score. No other operative management factors were significantly associated with status at 18 months of age.

**Table 3:** Single and multiple covariate risk factor models for neurodevelopmental outcomes (N = 110).

Potential Risk Factors	Cognition					Language					Motor				
	Single Covariate			Best Fitting Multiple Covariate		Single Covariate			Best Fitting Multiple Covariate		Single Covariate			Best Fitting Multiple Covariate	
	*n*	β (SE)	*p*	β (SE)	*p*	*n*	β (SE)	*p*	β (SE)	*p*	*n*	β (SE)	*p*	β (SE)	*p*
*Maternal Characteristics*															
Gestational diabetes	110	2798.5 (4023.3)	0.49			110	10.7 (9.1)	0.24			110	12778.9 (42294.9)	0.76		
Gestational hypertension	110	-2779.4 (7948.1)	0.73			110	-21.1 (18.0)	0.24			110	-58803.9 (83263.6)	0.48		
Smoke exposure	110	-3369.8 (2605.8)	0.20			110	-0.5 (6.0)	0.93			110	-420.9 (27551.8)	0.99		
Preeclampsia	110	8255.6 (4566.8)	0.07			110	2.4 (10.5)	0.82			110	-153.0 (48629.3)	1.00		
Impaired Maternal-Fetal															
Environment	110	99.0 (2040.2)	0.96			110	1.5 (4.6)	0.74			110	-882.5 (21410.0)	0.97		
Maternal age	110	330.1 (132.5)	0.01			110	0.5 (0.3)	0.11			110	2780.3 (1404.4)	0.05		
Multiple Gestation	110	-5128.8 (3053.2)	0.09			110	-13.8 (6.9)	0.04			110	-34102.4 (32284.3)	0.29		
Placental weight (kg)	89	3.8 (6.6)	0.56			89	0.0 (0.0)	0.47			89	64.0 (65.5)	0.33		
Placental wt./birth wt.	89	-10471.8 (19410.0)	0.59			89	-28.9 (44.1)	0.51			89	-67434.8 (192003.8)	0.72		
Maternal education	110		**0.01**			110		**< 0.01**			110		0.15		
College graduate		-2101.6 (1751.2)	0.23				-4.4 (3.9)	0.25				-9250.3 (19011.4)	0.63		
High School diploma		-6932.2 (2733.1)	0.01				-19.6 (6.0)	**< 0.01**				-29937.1 (29671.8)	0.31		
Partial college		-5820.3 (2325.1)	0.01				-18.0 (5.1)	**< 0.01**				-49541.2 (25241.7)	0.05		
Partial high school		-9642.3 (3337.6)	**< 0.01**				-19.0 (7.4)	0.01				-68439.3 (36234.0)	0.06		
Graduate school (ref)															
*Patient Characteristics*															
Preterm birth	110	-4400.9 (2721.7)	0.10			110	-11.6 (6.2)	0.06			110	-58128.6 (28361.6)	**0.04**		
Gestational age	110	1123.6 (585.6)	0.06	5290.9 (1875.0)	**< 0.01**	110	3.4 (1.3)	**0.01**			110	14504.2 (6092.0)	**0.02**		
Small for gestational age	110	-6367.6 (2257.9)	**< 0.01**			110	-12.8 (5.2)	**0.01**			110	-59415.9 (23872.4)	**0.01**		
Hollingshead Raw Score	109	227.7 (53.9)	**< 0.01**	3856.0 (1577.6)	**0.01**	109	0.5 (0.1)	**< 0.01**	0.4 (0.1)	**< 0.01**	109	1217.0 (594.3)	0.04		
Sex	110	-4290.3 (1498.8)	**< 0.01**			110	-13.2 (3.3)	**< 0.01**	-12.6 (2.9)	**< 0.01**	110	-72867.1 (14748.7)	**< 0.01**	-62926.5 (12852.2)	**< 0.01**
Race	110		**0.01**			110		**0.01**			110		0.01		
African American		-9336.8 (3213.2)	**< 0.01**				-21.3 (7.3)	**< 0.01**				-10274.0 (33595.1)	**< 0.01**		
Other		-1817.0 (2126.3)	0.39				-6.4 (4.8)	0.18				-18473.3 (22231.1)	0.41		
White (ref)															
Ethnicity	110	-4716.8 (2093.1)	0.02			110	-14.8 (4.6)	**< 0.01**			110	-61878.6 (21677.5)	**< 0.01**	-43781.0 (17940.2)	0.01
Cardiac diagnosis	110		0.40			110		0.26			110		0.02		
HLHS (ref)															
TGA		1644.3 (1872.6)	0.38				3.9 (4.2)	0.36				55386.2 (19081.1)	**< 0.01**		
Other		-714.4 (1872.6)	0.70				- 2.8 (4.2)	0.51				23480.6 (19081.1)	0.22		
Genetic anomaly	110		**0.01**		**0.01**	110		**< 0.01**		**< 0.01**	110		**< 0.01**		**0.01**
Confirmed		-3480.7 (1979.5)	0.08	-2192.8 (1825.90)	0.23		- 12.6 (4.3)	**< 0.01**	-8.1 (3.9)	**0.04**		-62607.2 (19960.6)	**< 0.01**	-47426.2 (17225.1)	**< 0.01**
Suspected		-6461.8 (2197.3)	**< 0.01**	-6653.9 (2093.1)	**< 0.01**		- 18.8 (4.8)	**< 0.01**	-15.7 (4.4)	**< 0.01**		-82288.3 (22156.6)	**< 0.01**	-46482.9 (20301.4)	**0.02**
APOE Genotype	107		0.43			107		0.89			107		0.67		
e2 (ε2ε2, ε2ε3)		-760.2 (2919.0)	0.79				2.9 (6.7)	0.66				26660.7 (31123.3)	0.39		
e3 (ε3ε3; ref)															
e4 (ε3ε4, ε4ε4)		2331.6 (1899.6)	0.22				1.1 (4.4)	0.80				-2381.8 (20254.8)	0.91		
Weight difference	110	-11.3 (675.7)	0.99			110	- 0.4 (1.5)	0.81			110	7761.3 (7052.2)	0.27		
Length difference	110	414.5 (542.5)	0.44			110	1.8 (1.2)	0.14			110	9618.6 (5633.8)	0.09		
Head circumference difference	110	320.1 (551.2)	0.56			110	0.8 (1.2)	0.51			110	6208.3 (5762.8)	0.28		
*Operative Management*															
Age at first operation	110	-19.4 (247.4)	0.94			110	-0.3 (0.6)	0.57			110	-2495.4 (2585.8)	0.33		
Operative class	110		0.28			110		0.50			110		0.06		
1v No Obstruction		-2330.1 (2951.4)	0.43				-6.9 (6.8)	0.31				-35206.2 (30470.9)	0.25		
1v Obstruction		54.9 (1742.0)	0.97				-0.2 (4.0)	0.95				-39698.6 (17984.6)	0.03		
2v No Obstruction (ref)															
2v Obstruction		3482.0 (2168.8)	0.11				4.7 (5.0)	0.35				15350.8 (22391.8)	0.49		
Total support time	110	-51.1 (23.1)	**0.03**			110	-0.1 (0.0)	0.15			110	-270.7 (246.2)	0.27		
Length of stay	110	-67.3 (20.8)	**< 0.01**			110	-0.1 (0.0)	0.02			110	-833.8 (214.0)	**< 0.01**	-572.3 (189.9)	**< 0.01**
*After First Operation*															
Additional Operations	110	-1681.3 (562.0)	**< 0.01**			110	-3.4 (1.3)	0.01			110	-21789.1 (5770.5)	**< 0.01**		
Additional Operations with CPB	110	-1091.1 (1537.2)	0.48			110	-1.8 (3.5)	0.61			110	-36161.8 (15795.9)	0.02		
Gestational															
age*Hollingshead															
Raw Score				-94.92 (40.89)	**0.02**										
Intercept	109			-18424.0 (72137.4)		109	82.1 (5.7)				110			332940.4 (11023.4)	

*Note*. All 0.0 values appearing here represent rounded values to retain 1 decimal place.

Urine concentration of exposure biomarkers were measured at 18 months of age; and blood levels were measured for lead, mercury, nicotine, cotinine, and hydroxycotinine. A urine sample was not available for 3 subjects. A blood sample was not available for 20 subjects. Mercury, nicotine, cotinine, and hydroxycotinine were detected in only 1–2 subjects each and these results were not included in the analyses. A summary of the analyte distributions along with their geometric means, standard errors, and corresponding percentiles is presented in S1 Table.

A Spearman-rho correlation matrix of the urinary metabolites concentrations was computed. Of the 870 bivariate correlations, 86 (10%) were highly, statistically significant (p ≤ 0.0024, with many correlations ranging from moderate (r = 0.40 to 0.60) to strong (r > 0.60 to 0.97). Phthalate metabolites in particular were more frequently and strongly correlated with one other urinary metabolite. These observations suggest that there are likely common sources for certain groups of contaminants. In future analyses, we will determine whether specific exposure profiles reflect unique patterns of device use (S2 Table).

Detectable concentrations of some pesticides [4-fluoro-3-phenoxybenzoic acid, trans-3-(2,2-dichlorovinyl)-2,2-dimethylcyclopropane carboxylic acid, and 2-isopropyl-4-methyl-6-hydroxypyrimidine] were found in only 6, 15, and 26 subjects respectively; and were therefore excluded from analysis. Measurable concentrations of *para*-nitrophenol, a nonspecific metabolite of parathion were detected in 106 subjects. Parathion is no longer used and there are other substances which can confound the measurements, so *para*-nitrophenol was excluded from further analysis. Very high concentrations of methyl- and ethyl parabens were detected in some subjects. These chemicals are commonly used in diaper creams. Because of concern over contamination during urine sample collection, these biomarkers were excluded from the analysis. To provide context about the magnitude of exposures compared to the general population, the biomarkers included in the final analysis were compared to available data from two published studies [40, 41] (S3 Table). The exposures for the current study were similar to previously reported data.

**Fig 2:**
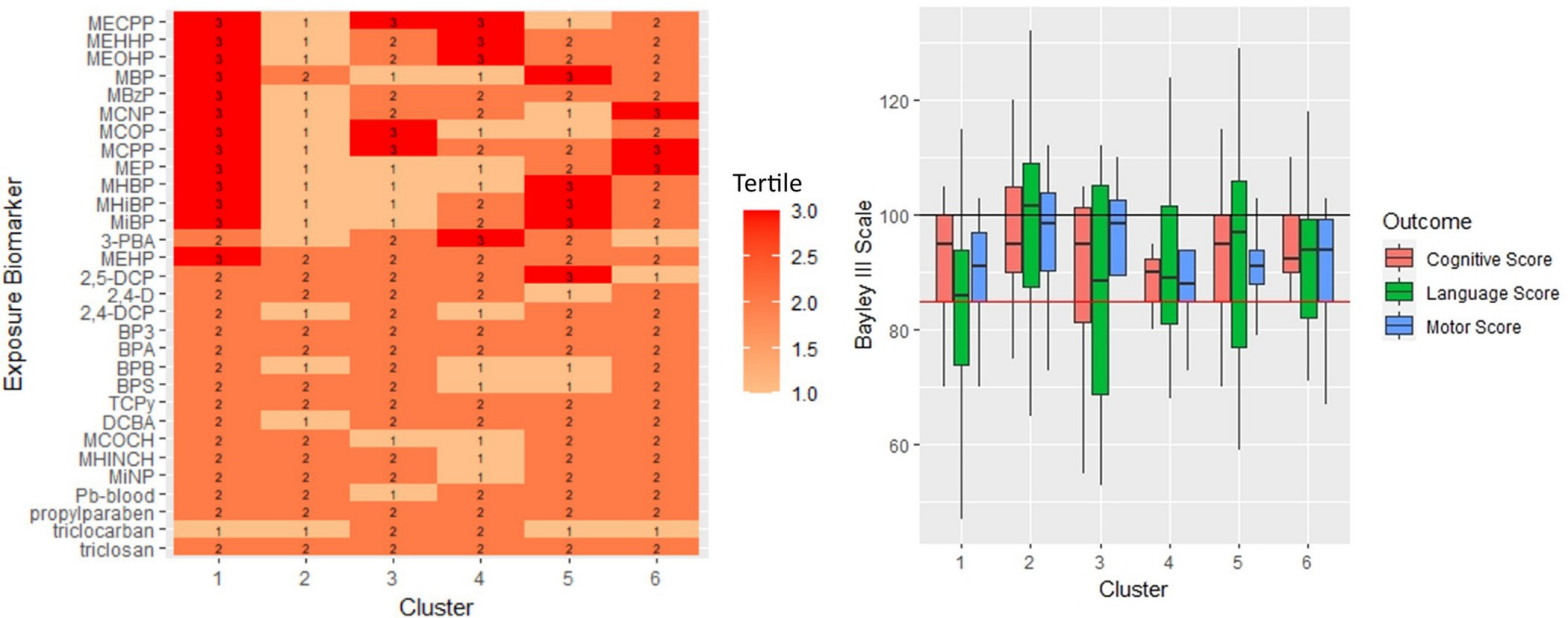
A) Exposure Biomarkers by Clusters: Heatmap to visualize the median (most typical) tertile of exposure that each chemical falls within for each cluster. Darker red colors indicate tertile 3 (highest exposure group), orange colors indicate tertile 2 (medium exposure group), and light orange indicate tertile 1 (lowest exposure group). B) Unadjusted Neurodevelopmental Scores by Cluster: Box and whisker plots of neurodevelopmental scores (unadjusted) for each cluster. Black lines within each boxplot represent the median value and the upper and lower ends of the boxes represent the 75th and 25th percentiles, respectively. The horizontal red line on the graph indicates 1 standard deviation (85) below population mean (100).

With BPR, we uniformly analyzed 30 chemical biomarkers, including urinary phenols, parabens, triclocarban and metabolites of pesticides and phthalates, and one blood biomarker (Pb) for the 110 subjects who returned for the 18-month evaluation. (Table 1) Three returnees were removed from the BPR analysis due to lack of any biomarker information, resulting in a final population for profile regression analysis of *n* = 107. Because profile regression can handle missing values in the exposures of interest, we were able to analyze the data of all 107 participants despite several missing values on a select few biomarkers of exposure (e.g., Pb). Six clusters were identified from BPR analysis of 30 environmental chemical biomarkers. In terms of number of subjects, cluster 1 is the largest cluster (*n* = 29), followed by cluster 2 (*n* = 28), cluster 5 (*n* = 21), cluster 6 (*n* = 18), cluster 4 (*n* = 7), and cluster 3 (*n* = 4). Cluster 1 represents the highest exposure group, with concentrations for 13 phthalate biomarkers that fall within the highest concentration tertile (Fig 2A). Conversely, cluster 2 represents the lowest exposure group with nearly all of the biomarkers involved in the clustering being in the lowest concentration tertile. All other clusters are characterized by elevated concentrations for only a few of the biomarkers involved in the clustering. The variable selection procedure indicated that 15 of the 30 chemical biomarkers drove the observed clustering pattern, 13 of which are phthalate metabolites. The only non-phthalate chemicals included in the clustering were a pyrethroid pesticide metabolite, 3-phenoxybenzoic acid (3-PBA) and a phenol, 2,5-dichlorophenol. There was no statistically significant variability among clusters in terms of study population characteristics (S4 Table). The clear partitioning of phthalate exposure biomarker profiles between the six clusters is shown in the heatmap (Fig 2A), suggesting that the BPR clustering algorithm successfully grouped together individuals with similar exposure biomarker profiles and separated those with dissimilar profiles. The full posterior joint distribution of the exposure biomarker profile clusters (with uncertainty) is provided in the Supplemental Materials (S1 Fig).

**Fig 3:**
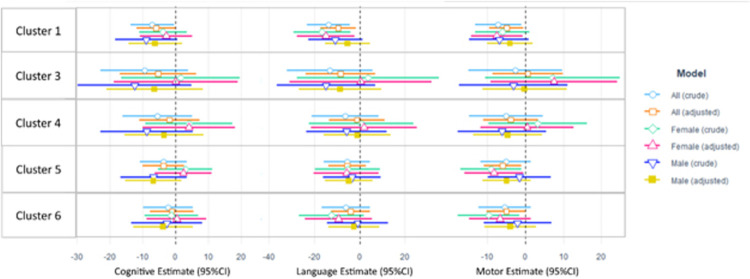
Assessment scores from linear regression models. Crude and adjusted linear regression models for the three outcomes of interest and the clusters from the profile regression. Cognitive Score Model adjusted for genetic anomaly, gestational age, and raw Hollingshead SES score. Language Score Model adjusted for genetic anomaly, raw Hollingshead SES score, and sex. Motor Score Model adjusted for ethnicity, genetic anomaly, sex, and hospital length of stay at first operation. Cluster 2 is the lowest exposure cluster and is set as the reference group for each model.

Boxplots of the unadjusted neurodevelopmental outcomes for each cluster are shown in Fig 2B. Cluster 1 (highest exposure group) had lower median Language and Motor Composite scores compared to cluster 2 (lowest exposure group). The distribution of the Language Composite scores for cluster 1 is shifted well below 1 standard deviation (85) from the population mean, with 41.4% of children in cluster 1 having a Language Composite score ≤ 85 and just 20.7% having a Language Composite score ≥ 100. Conversely, cluster 2 consistently resulted in the highest median scores for Language and Motor Composite scores, with only 17.9% having a Language Composite score ≤ 85 and 53.6% having a Language Composite score ≥ 100. Crude and adjusted Cognitive, Language, and Motor Composite scores for the cohort and stratified by clusters are shown in Table 4 and Fig 3. Overall, there were no statistically significant differences for Cognitive Composite scores between the clusters and the reference group, cluster 2 (lowest exposure). Relative to cluster 2, children in cluster 1 (highest exposure group) had significantly lower Language Composite scores by -9.41 points (95%CI: -17.2, -1.7) and significantly lower Motor Composite scores by -4.9 points (-9.5, -0.4) after adjustment. The Motor Composite score was significantly lower for cluster 5 compared to cluster 2 by -5.91 points (-10.85, -0.97). Models stratified by sex indicated that these negative exposure-response relationships for cluster 1 were only statistically significant among girls. Girls in cluster 1 had significantly lower Language Composite scores by -14.9 points (-27.7, -2.2) compared to cluster 2, whereas the negative association was attenuated in boys (β = -5.3 [-15.3, 4.7]) in the adjusted models. A similar sex-specific pattern was observed for the Motor Composite scores, whereby cluster 1 was significantly associated with lower scores among girls (β = -7.5 [-14.3, -0.7]) but not among boys (β = -4.0 [-10.0, 2.0]) in the adjusted models. Cluster 5 also exhibited a significant adjusted association for Motor Composite scores among girls only, with girls in cluster 5 having a lower score by -8.2 points (-16.0, -0.5) compared to girls in cluster 2.

**Table 4:** Crude and adjusted neurodevelopmental outcomes by cluster.

**Cognitive Score**	** **	** **	** **	** **	** **	** **	** **	** **	** **	** **	** **	** **	** **	** **	** **	** **	** **	** **
	**Overall (Crude)**			**Overall (Adjusted)**			**Females (Crude)**			**Females (Adjusted)**			**Males (Crude)**			**Males (Adjusted)**		
*Predictors*	*Estimates*	*(CI)*	*p*	*Estimates*	*(CI)*	*p*	*Estimates*	*(CI)*	*p*	*Estimates*	*(CI)*	*p*	*Estimates*	*(CI)*	*p*	*Estimates*	*(CI)*	*p*
Cluster 1	-7.07	(-13.71, -0.42)	**0.037**	-5.84	(-11.73, 0.04)	0.052	-3.79	(-11.05, 3.48)	0.307	-2.84	(-10.85, 5.17)	0.488	-8.87	(-18.36, 0.62)	0.067	-6.15	(-14.29, 1.99)	0.139
Cluster 3	-9.39	(-22.80, 4.02)	0.17	-5.23	(-16.95, 6.49)	0.382	1.67	(-16.45, 19.78)	0.857	0.14	(-18.76, 19.04)	0.988	-12.48	(-29.85, 4.89)	0.159	-6.34	(-21.10, 8.43)	0.4
Cluster 4	-5.46	(-16.07, 5.14)	0.312	-1.79	(-11.05, 7.47)	0.705	4.17	(-9.12, 17.46)	0.539	4.18	(-9.89, 18.25)	0.56	-8.81	(-22.96, 5.34)	0.222	-3.41	(-15.44, 8.61)	0.578
Cluster 5	-3.65	(-10.90, 3.59)	0.323	-3.68	(-9.98, 2.62)	0.252	3.1	(-5.18, 11.37)	0.464	2.42	(-6.24, 11.07)	0.584	-6.67	(-16.78, 3.44)	0.196	--6.73	(-15.27, 1.82)	0.123
Cluster 6	-2.17	(-9.75, 5.41)	0.575	-1.11	(-7.71, 5.49)	0.742	-1.19	(-9.47, 7.09)	0.778	0.39	(-8.67, 9.45)	0.933	-2.63	(-13.45, 8.19)	0.634	-3.76	(-12.77, 5.26)	0.414
Presence of Genetic Anomaly				-5.32	(-8.42, -2.22)	**0.001**				-2.25	(-6.93, 2.43)	0.346				-5.72	(-9.89, -1.54)	**0.007**
Older Gestational Age				9.88	(3.71, 16.06)	**0.002**				-5.45	(-23.88, 12.98)	0.562				8.64	(1.31, 15.96)	**0.021**
Higher Hollingshead Raw Score				6.99	(1.79, 12.18)	**0.008**				-4.07	(-18.67, 10.53)	0.585				6.4	(0.30, 12.50)	**0.04**
Gestational Age * Hollingshead Raw Score				-0.17	(-0.31, -0.04)	**0.011**				0.11	(-0.27, 0.48)	0.577				-0.16	(-0.32, 0.00)	0.054
Observations	107			107			40			40			67			67		
R^2^	0.052			0.323			0.09			0.145			0.076			0.404		
**Language Score**	** **	** **	** **	** **	** **	** **	** **	** **	** **	** **	** **	** **	** **	** **	** **	** **	** **	** **
	**Overall (Crude)**			**Overall (Adjusted)**			**Females (Crude)**			**Females (Adjusted)**			**Males (Crude)**			**Males (Adjusted)**		
*Predictors*	*Estimates*	*(CI)*	*p*	*Estimates*	*(CI)*	*p*	*Estimates*	*(CI)*	*p*	*Estimates*	*(CI)*	*p*	*Estimates*	*(CI)*	*p*	*Estimates*	*(CI)*	*p*
Cluster 1	-13.71	(-23.03, -4.40)	**0.004**	-9.41	(-17.15, -1.68)	**0.017**	-16.52	(-29.08, -3.97)	**0.01**	-14.94	(-27.67, -2.22)	**0.021**	-10.66	(-22.50, 1.18)	0.078	-5.3	(-15.31, 4.70)	0.299
Cluster 3	-13.14	(-31.94, 5.65)	0.171	-8.38	(-23.8, 7.04)	0.287	3.75	(-27.56, 35.06)	0.814	0.52	(-30.75, 31.80)	0.974	-14.77	(-36.45, 6.91)	0.182	-8.5	(-26.65, 9.66)	0.359
Cluster 4	-6.36	(-21.22, 8.50)	0.402	-1.21	(-13.46, 11.04)	0.846	0.75	(-22.23, 23.73)	0.949	1.92	(-21.25, 25.08)	0.871	-5.84	(-23.50, 11.82)	0.517	-0.91	(-15.66, 13.83)	0.903
Cluster 5	-5.6	(-15.75, 4.56)	0.28	-5.41	(-13.76, 2.94)	0.204	-5.25	(-19.56, 9.06)	0.472	-5.81	(-20.07, 8.44)	0.424	-3.37	(-15.98, 9.25)	0.601	-4.68	(-15.19, 5.84)	0.383
Cluster 6	-6.03	(-16.65, 4.59)	0.266	-3.99	(-12.67, 4.69)	0.367	-12.39	(-26.70, 1.91)	0.09	-9.22	(-23.94, 5.50)	0.22	-0.89	(-14.39, 12.61)	0.897	-2.51	(-13.70, 8.68)	0.66
Male Sex				-13.18	(-18.99, -7.38)	**<0.001**												
Hollingshead Raw Score				0.41	(0.19, 0.63)	**<0.001**				0.28	(-0.13, 0.69)	0.179				0.47	(0.20, 0.74)	**0.001**
Presence of Genetic Anomaly				-7.38	(-11.44, -3.33)	**<0.001**				-2.74	(-10.49, 5.01)	0.488				-9.37	(-14.32, -4.43)	**<0.001**
Observations	107			107			40			40			67			67		
R^2^	0.081			0.408			0.203			0.263			0.073			0.386		
**Motor Score**	** **	** **	** **	** **	** **	** **	** **	** **	** **	** **	** **	** **	** **	** **	** **	** **	** **	** **
	**Overall (Crude)**			**Overall (Adjusted)**			**Females (Crude)**			**Females (Adjusted)**			**Males (Crude)**			**Males (Adjusted)**		
*Predictors*	*Estimates*	*(CI)*	*p*	*Estimates*	*(CI)*	*p*	*Estimates*	*(CI)*	*p*	*Estimates*	*(CI)*	*p*	*Estimates*	*(CI)*	*p*	*Estimates*	*(CI)*	*p*
Cluster 1	-7.14	(-13.24, -1.04)	**0.022**	-4.94	(-9.46, -0.42)	**0.032**	-6.02	(-13.13. 1.08)	0.097	-7.5	(-14.30, -0.69)	**0.031**	-6.9	(-14.73, 0.93)	0.084	-4.01	(-10.00, 1.98)	0.19
Cluster 3	-2.61	(-14.92, 9.70)	0.678	0.65	(-8.53, 9.83)	0.89	7.25	(-10.47, 24.97)	0.423	7.54	(-9.06, 24.15)	0.373	-3.13	(-17.46, 11.21)	0.669	-0.18	(-11.28, 10.91)	0.974
Cluster 4	-5.11	(-14.84, 4.63)	0.304	-3.82	(-11.08, 3.43)	0.302	3.25	(-9.75, 16.25)	0.624	0.49	(-11.8, 12.78)	0.938	-6.13	(-17.80, 5.55)	0.304	-4.63	(-13.65, 4.39)	0.314
Cluster 5	-5.15	(-11.80, 1.49)	0.129	-5.91	(-10.85, -0.97)	**0.019**	-9.04	(-17.13, -0.94)	**0.029**	-8.23	(-15.98, -0.48)	**0.037**	-1.55	(-9.89, 6.78)	0.715	-4.88	(-11.26, 1.49)	0.133
Cluster 6	-5.44	(-12.4, 1.82)	0.125	-5.02	(-10.20, 0.16)	0.058	-9.61	(-17.70, -1.51)	**0.02**	-6.67	(-14.78, 1.44)	0.107	-2.03	(-10.96, 6.89)	0.655	-3.98	(-10.79, 2.84)	0.253
Male Sex				-7.93	(-11.38, -4,48)	**<0.001**												
Hispanic Ethnicity				-5.06	(-9.84, -4.48)	**0.038**				-2.65	(-10.78, 5.47)	0.522				-6.07	(-12.15, 0.01)	0.05
Presence of Genetic Anomaly				-4.36	(-6.89, -1.83)	**0.001**				-0.06	(-4.27, 4.15)	0.977				-5.62	(-8.85, -2.38)	**0.001**
Longer Length of Stay First Hospitalization				-0.01	(-0.15, -0.05)	**<0.001**				-0.33	(-0.57, 0.010)	**0.006**				-0.08	(-0.13, -0.03)	**0.003**
Higher Birth Weight z-Score				2.47	(0.73, 4.22)	**0.005**				0.19	(-3.08, 3.46)	0.909				3	(0.89, 5.10)	**0.005**
Observations	107			107			40			40			67			67		
R^2^	0.055			0.513			0.25			0.427			0.058			0.496		
Cluster 2 is Reference																		

**Fig 4:**
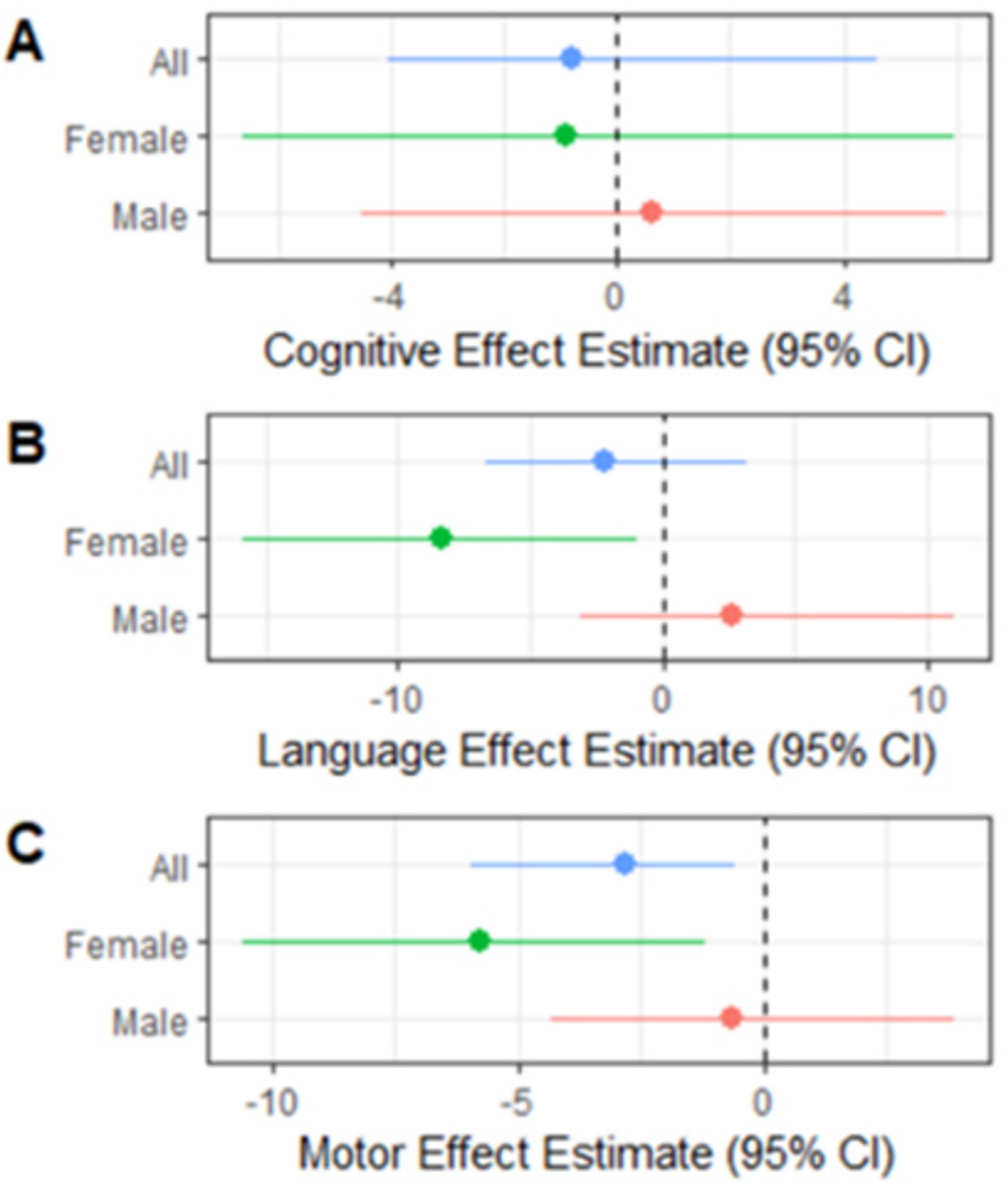
Weighted quantile sum (WQS) regression effect estimate. The WQS modeling results for selected neurodevelopmental outcomes displaying the mean and lower 2.5^th^ and upper 97.5^th^ percentiles for the WQS index coefficients obtained from the repeated holdout validation.

**Fig 5:**
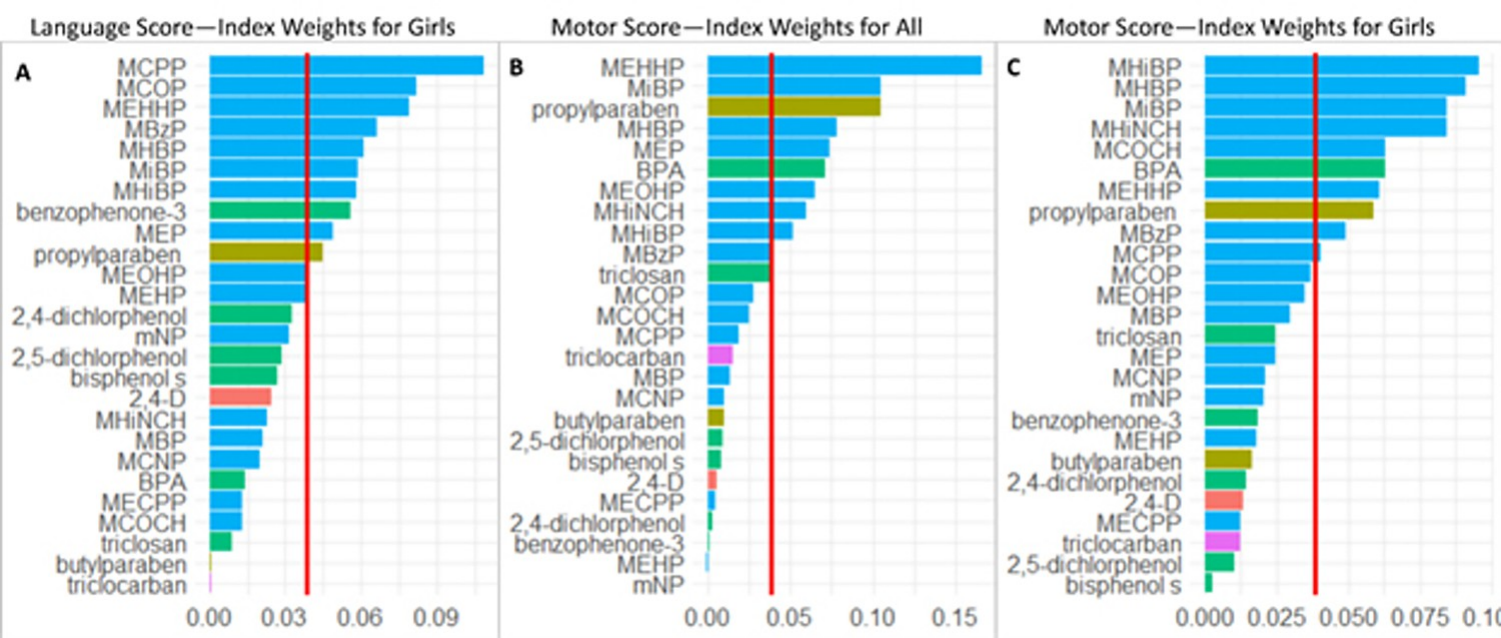
Weighted quantile sum regression. Index weights for WQS index exposure-responses that were significantly associated with ND outcomes. (A) Language score index weights for girls; (B) Motor score index weights for entire cohort; (C) Motor score index weights for girls.

Complete exposure data were available for 26 urinary biomarkers among 107 children and were included in the WQS analysis. The WQS modeling results for selected neurodevelopmental outcomes are shown in Fig 4, which displays the mean and lower 2.5^th^ and upper 97.5^th^ percentiles for the WQS index coefficients obtained from the repeated holdout validation. Overall, only the Motor Composite score resulted in a significantly lower score (β = -2.8 points [2.5^th^ and 97.5^th^ percentile: -6.0, -0.6]) for the exposure-response relationship of the overall mixture index. The female-only WQS models resulted in significantly lower Language Composite score by -8.4 points (2.5^th^ and 97.5^th^ percentile: -15.9, -0.9) and lower Motor Composite score by -5.7 points (2.5^th^ and 97.5^th^ percentile: -10.6, -1.2) for exposure-response of the overall mixture index. No significant association were observed in male-only WQS models (Fig 5). The WQS index weights for each of the 26 exposure biomarkers included in the analysis are shown in Fig 5. For Motor Composite score among all participants (Fig 5B), several phthalate metabolites mono-2-ethyl-5-hydroxyhexyl phthalate (MEHHP) [16.6%], mono-isobutyl phthalate (MiBP) [10.5%], mono-hydroxybutyl phthalate (MHBP) [7.8%], mono-ethyl phthalate (MEP) [7.4%], mono-2-ethyl-5-oxohexyl phthalate (MEOHP) [6.5%], and mono-hydroxyisobutyl phthalate (MHiBP) [5.1%], one metabolite of a phthalate alternative, cyclohexane-1 2-dicarboxylic acid monohydroxy isononyl ester (MHINCH) [5.9%], one paraben (propylparaben [10.5%]), and one phenol, bisphenol A (BPA) [7.1%] resulted in index weights that suggest they are important contributors to the outcomes (probability> 1/26 [3.8%]). For Language Composite score among girls (Fig 4A), several phthalates mono-3-carboxypropyl phthalate (MCPP) [10.9%], mono carboxyisooctyl phthalate (MCOP) [8.2%], MEHHP [7.9%], monobenzyl phthalate (MBzP) [6.6%], MHBP [6.1%], MiBP [5.9%], MHiBP [5.8%], and MEP [4.9%]), one phenol (benzophenone-3 [5.6%]), and propylparaben [4.5%]) resulted in index weights that suggested they are important biomarkers driving the negative exposure-response relationship of the WQS index. Similarly, for Motor Composite scores among girls only (Fig 5C), several phthalates (MHiBP [9.5%], MHBP [9.1%], MiBP [8.5%], MEHHP [6.1%], MBzP [4.9%], and MCPP [4.0%]), two metabolites of a phthalate alternative (MHINCH [8.4%], and cyclohexane-1 2-dicarboxylic acid monocarboxyisooctyl ester (MCOCH) [6.3%]), one phenol (BPA [6.3%]) and propylparaben [5.9%]) resulted in index weights that suggest they contributed significantly to the overall WQS index.

## Discussion

Exposure to potentially neurotoxic chemicals during susceptible periods of rapid brain growth can have profound effects on neurodevelopment.[4, 42] In the current study, we demonstrate that toddlers with CHD are exposed to complex mixtures of chemicals (e.g., phthalates, phenols, parabens, pesticides), some of which are known or suspected EDCs and neurotoxicants, during everyday life. There is significant variability in both the pattern and magnitude of exposures; however, the magnitude of exposure is similar to that reported for infants and toddlers in the general population [40, 41]. Greater concentrations of biomarkers of exposure to these chemicals, especially phthalates, are associated with poorer performance for language and motor skills at 18 months of age after adjustment for known risk factors for adverse neurodevelopment outcomes. Many of these chemicals are known EDCs and/or neurotoxicants and we identified a greater adverse effect in girls compared to boys. This study adds to the growing body of evidence of the importance of patient and environmental factors, such as fetal brain development, genetic syndromes, and SES, as determinants of neurodevelopmental outcomes in the CHD population. In order to define factors that may predispose to, or protect against, brain injury and adverse neurodevelopmental outcomes in the CHD population, we must consider the neurodevelopmental exposome, i.e., the *totality* of exogenous and endogenous exposures from conception onward through adult life [43].

Exposure to phthalates, both in utero and later in life, has been associated with neurobehavioral disability. A systematic review of the literature by Ejaredar and associates found evidence that prenatal exposure to phthalates is associated with adverse cognitive and behavioral outcomes, including lower IQ, and problems with attention, hyperactivity, and poorer social communication [44]. In an important study, Verstraete and colleagues investigated the relationship of circulating phthalate metabolites in a cohort of more than 400 critically ill children (ages 0 to 16 years) to subsequent development of attention deficit hyperactivity disorder (ADHD) compared with normal controls [45]. The phthalate exposure explained half of the risk of attention deficit in the cohort. They concluded that “Iatrogenic exposure to DEHP metabolites during intensive care was independently and robustly associated with the important attention deficit observed in children 4 years after critical illness” [45]. In a study of early life phthalate exposures, Li and coworkers found evidence that childhood exposures to phthalate mixtures was associated with increased behavior problems [46].

In both the cluster analysis and the WQS regression analysis, we found that exposure to mixtures of multiple chemical classes (e.g., phthalates, pesticides, phenols, parabens) was associated with worse outcomes. These classes of chemicals have been associated with adverse neurodevelopmental outcomes in other populations [7, 10, 47]. The most commonly identified exposure was to phthalates [diesters of phthalic acid] used as solvents and plasticizers (to give flexibility) in many types of products such as food packaging, cosmetics, medical devices, toys, dentures, paints, adhesives, and nail polishes. Phthalates are not covalently bound and thus handling these products can lead to significant exposures [41]. In addition, phthalates and pesticides are commonly found in household dust [11, 12]. Prenatal and early childhood exposures to phthalates have been associated with poorer neurobehavioral outcomes [6, 48]. After exposure, phthalate diesters are rapidly metabolized and excreted in the urine. Urinary concentrations of these metabolites can be utilized as exposure biomarkers and reflect recent exposures (half-lives of 6 to 24 hours). Di(2-ethylhexyl) phthalate (DEHP) is one of the most commonly used phthalates. Four DEHP metabolites (MECPP, MEHHP, MEOHP, MEHP) were included in the high exposure group (cluster 1); both MEHHP and MEOHP were identified in the WQS regression as important drivers of the adverse exposure-response relationship. Metabolites of several other phthalates were associated with poorer outcomes by both the cluster analysis and WQS regression, including metabolites of di-isobutyl phthalate (MHiBP), benzylbutyl phthalate (MBzP), dibutyl phthalate (MHBP), di-n-octyl phthalate (MCPP), and diethyl phthalate (MEP). Non-phthalate chemicals were also important in the clustering including a pyrethroid pesticide metabolite and 2,5-dichlorophenol. The WQS regression also identified exposure biomarkers of other classes of chemicals (phenols and parabens) as potential drivers of adverse outcomes.

As noted in a recent review, “The staggering majority of epidemiological studies have investigated chemicals in isolation rather than taking into account the totality of chemical exposures, which together may have additive, synergistic, antagonistic, or potentiating effects.” [42] A major strength of our study is the use of statistical modeling approaches specifically designed to assess the challenges of analyzing the health effects of multiple correlated environmental exposures. Epidemiologic modeling of the health effects of chemical mixtures, and especially EDCs, must deal with the large number of possible exposures and the potentially highly correlated (multi-collinearity) nature of exposure to multiple EDCs. Large numbers of co-exposures and multi-collinearity make it particularly challenging in terms of being able to disentangle causal effects of particular EDCs within the chemical mixture. Consequently, in this study, we utilized statistical alternatives to multivariable regression that have been developed by others to deal with such challenges [36, 39, 49–52]. Specifically, we applied cluster analysis using BPR as one approach that groups observations together based on their similarity in ‘exposure biomarker profiles’. Grouping individuals based on similarity in exposure biomarkers can serve as a type of dimension reduction technique for evaluating health effects of chemical mixtures. Cluster analysis also helps to identify typical exposure biomarker patterns in a study population which can identify sub-populations of individuals who are most vulnerable to multiple exposures and susceptible to adverse health effects. WQS regression is another technique applied in our study that was developed as a way to estimate both an overall effect of the chemical mixture and to derive an exposure-response index weighted by the relative contribution that a particular chemical exposure has to health outcomes of interest (response). This approach therefore helps to identify possible chemicals with worse potential adverse health effects among an array of chemical exposures while also estimating the adverse effects of the mixture. In the current study, we applied BPR (a type of cluster analysis) and WQS to deal with multi-collinearity and for dimension reduction in the exposure-response space. These methods have been applied in other recent studies evaluating the health effects of chemical mixtures in children, including neurodevelopmental outcomes [6, 15–17, 36, 53–55]. In our study, we found these distinct statistical methods to be complementary, insofar as cluster analysis describes sub-populations in terms of their exposures to multiple chemicals and associated adverse neurodevelopmental risks, whereas WQS is concerned with assessing the overall mixture effects on neurodevelopmental risks as well as highlighting specific chemical exposures that may be driving such risks. Another strength is the detailed characterization of the cohort in terms of known and suspected risk factors for poor neurodevelopmental outcomes.

Previous studies have shown that the associations of select chemical exposures with neurodevelopmental outcomes are mixture and sex specific [6, 56, 57]. The mechanisms resulting in sex differences have not been fully delineated. Many of these chemicals are known EDCs, which can have differential effects on girls and boys due to their different hormone profiles [57]. EDCs are defined as exogenous chemicals that can interfere with any aspect of hormone action [57]. The potential mechanism of EDC mediated neurotoxicity is disruption of endocrine signaling, mimicking the actions of thyroid, glucocorticoid, and gonadal hormones [3, 42].

Because of the increasing recognition of the toxicity of EDCs, particularly phthalates, there have been efforts in some countries to use alternative plasticizers, including in medical equipment [58, 59]. A variety of alternative plasticizers have been utilized [59]. Studies have shown that there is often lower migration of the alternative plasticizers compared to those manufactured with DEHP, but the toxicity profiles (including impact on neurodevelopment) are not fully known [59, 60]. Substitution of alternative plasticizers for phthalates may not always improve outcomes [60]. In a study of NICU patients, use of DEHP-free central venous lines for total parenteral nutrition was associated with a significant decrease in cholestasis [61]. However, in a follow-up to the study evaluating phthalates and ADHD, phasing out DEHP containing devices was shown to reduce DEHP exposure, but there was still significant exposures and the incidence of impaired attention was not reduced [62].

There are limitations to this study. The cohort is relatively small which precluded us from to explicitly testing for statistical interaction effects of specific combinations of exposure biomarkers. While one interpretation of the BPR method could be that interactions are accounted for through effects of latent variables (clustering of exposure profiles), the method is not designed to estimate or test interaction effects between specific chemical exposures of interest. Therefore, BPR at best is an indirect way of assessing the possibility of interaction effects on an outcome. Despite the limitations driven by the relatively small study sample size, leveraging BPR is a strength of this study because it allows us to explore exposure-response relationships given specific combinations of biomarker exposure levels. Another limitation of our application of BPR is the use of “hard” clusters. There is inherent uncertainty in cluster allocation with any type of cluster analysis. While BPR propagates uncertainty by fitting with Markov chain Monte Carlo (MCMC) sampling methods, we focused our epidemiological inference on the deterministic clustering allocation. In other words, we ignored the uncertainty of cluster allocation and instead used the “hard groupings” (or best clustering) as determined with the optimal partition algorithm, “partitioning around medoids” (PAM). Despite this limitation, the hard clustering provides a representative and interpretable cluster allocation that allowed us to model the crude and adjusted associations between exposure profiles and the different neurological outcomes. The assessment of exposure was limited to a single time point and the analyses evaluated cross-sectional exposures and outcomes. While relying on a single exposure biomarker for short-lived compounds certainly leads to exposure misclassification, this misclassification bias will typically be non-differential, which entails effect estimates will be attenuated towards the null. Future studies should include exposure and outcome assessment at multiple time points, including peri-operative, across the life span. Although we assessed multiple classes of chemicals, we did not assess other potentially neurotoxic compounds, including air pollution, flame retardants, and heavy metals other than Pb and mercury. Additionally, our exposure data for Pb included several missing observations. Very little comparative data for the exposure biomarkers are available for 18-month-old children. We also note that two of the clusters (C3 and C4) include small numbers of children. While these two clusters did not show a significant association with neurodevelopmental outcomes, this observation may be driven by the small sample size. Additionally, WQS regression assumes homogeneity for the directionality of the exposure-response relationship. This assumption does not allow for the possibility that some short-lived EDCs could plausibly impart a positive effect on the neurodevelopmental outcomes. Although, our sensitivity analysis suggested there was not a significant positive effect of the mixture. Finally, the neurodevelopment assessment was performed early in life and may not be predictive of later outcomes.

In conclusion, we demonstrate that early life chemical exposures are potentially important factors which should be further investigated as an important contributor to neurodevelopmental disability in children with CHD. In addition, we show that environmental exposure to multiple potentially toxic chemicals and EDCs (e.g., phthalates, pesticides, phenols, parabens) is ubiquitous in toddlers with CHD. There is significant variability in both the pattern and magnitude of exposure biomarkers. Greater concentrations of exposure biomarkers are associated with poorer outcomes for language and motor skills, especially for girls. In order to define factors that may predispose to, or protect against, brain injury and adverse neurodevelopmental outcomes in the CHD population; we must consider not only events that occur during medical care, but the neurodevelopmental exposome, the totality of exogenous and endogenous exposures from conception onward through adult life [43].

## Supporting information

S1 Methods Whole exome sequencing. (DOCX)

S1 Table Detection rates, geometric means, and percentiles (*N* = 110). (DOCX)

S2 Table Intercorrelation matrix of analytes (*N* = 110). (DOCX)

S3 Table Comparison to existing population exposure data. (DOCX)

S4 Table Cohort characteristics (overall and stratified by cluster). (DOCX)

S1 Fig The posterior distribution of proportion of individuals falling into a tertile of exposure for each chemical, within each of the six clusters that were found. (DOCX)
