# Supplementary material for: Childhood exposures to environmental chemicals and neurodevelopmental outcomes in congenital heart disease
Source: PLoS One. 2022 Nov 17;17(11):e0277611. doi: 10.1371/journal.pone.0277611 (PMC9671412; doi:10.1371/journal.pone.0277611)
Supplement: S1 Methods — (DOCX) [file pone.0277611.s001.docx]

**Supplemental Files**

**Methods**

**Whole Exome Sequencing**

Whole exome sequencing was performed on subjects and all consented parents. Exons were captured from fragmented and adaptor ligated genomic DNA samples using the SureSelect Human All Exon v 5 containing 51 Mb (Agilent Technologies, Santa Clara, California). Paired-end 2 x 101-base massively parallel sequencing was carried out on the Illumina HiSeq2500 platform (Illumina, San Diego, California), according to the manufacture’s protocols. Base calling was performed by the Illumina CASAVA software (v.1.8.2) with default measures. Sequencing reads passing the quality filter were aligned to the human reference genome (GRCh37-derived alignment set used in 1000 Genomes Project) with Burrows-Wheeler Aligner (v.0.7.12) and Dragen (Illumina Inc, San Diego, California).(1) Polymerase chain reaction (PCR) duplicates were removed using Picard (v.1.97, Broad Institute, Boston, Massachusetts). The Genome Analysis Toolkit (v.2.6–5, Broad Institute, Boston, Massachusetts) was used to generate variant calls.(2)

1. Li H, Durbin R. Fast and accurate long-read alignment with Burrows-Wheeler transform. Bioinformatics. 2010;26(5):589-95.

2. DePristo MA, Banks E, Poplin R, Garimella KV, Maguire JR, Hartl C, et al. A framework for variation discovery and genotyping using next-generation DNA sequencing data. Nat Genet. 2011;43(5):491-8.
