# Supplementary material for: Childhood exposures to environmental chemicals and neurodevelopmental outcomes in congenital heart disease
Source: PLoS One. 2022 Nov 17;17(11):e0277611. doi: 10.1371/journal.pone.0277611 (PMC9671412; doi:10.1371/journal.pone.0277611)
Supplement: S1 Table — (DOCX) [file pone.0277611.s002.docx]

**S1 Table: Detection Rates, Geometric Means, and Percentiles (*N* = 110)**

Detection Rate < LOD Missing Imputed and Specific Gravity Adjusted

Geometric Mean and Percentile Values

Analyte *f* (%) *f* (%) *f* (%) Mean (SError) P_25_ P_50_ P_75_ P_90_

***Organophosphates***

24D 65 (59) 42 (38) 3 (3) 0.23 (0.02) 0.13 0.22 0.33 0.57

CPM 90 (82) 15 (14) 5 (4) 0.63 (0.07) 0.33 0.73 1.40 2.17

OPM 77 (70) 28 (25) 5 (5) 0.42 (0.06) 0.17 0.35 1.06 2.70

***Phenols, Parabens, Trichlocarbans***

24DCP 107 (97) 3 (3) 0.37 (0.04) 0.27 0.38 0.66 1.14

25DCP 107 (97) 3 (3) 0.48 (0.08) 0.21 0.46 1.18 2.34

BP3 107 (97) 3 (3) 32.38 (5.72) 8.42 19.15 86.34 535.47

BPA 107 (97) 3 (3) 2.80 (0.39) 0.90 2.40 6.14 17.07

BPB 107 (97) 3 (3) 0.04 (0.01) 0.00 0.09 0.29 1.50

BPS 107 (97) 3 (3) 0.99 (0.10) 0.46 0.85 1.68 3.66

PPB 107 (97) 3 (3) 11.83 (2.04) 2.57 9.05 43.20 121.97

TCC 107 (97) 3 (3) 0.01 (0.00) 0.00 0.04 0.14 0.30

TCS 107 (97) 3 (3) 3.00 (0.60) 1.48 3.48 8.51 25.17

***Phthalates***

MBP 107 (97) 3 (3) 17.11 (1.36) 9.77 15.88 27.62 53.22

MBZP2 107 (97) 3 (3) 4.30 (0.44) 1.85 4.54 7.64 17.81

MCNP 107 (97) 3 (3) 2.06 (0.18) 1.26 1.98 3.17 5.67

MCOCH 107 (97) 3 (3) 0.56 (0.11) 0.44 0.78 1.53 3.49

MCOP 107 (97) 3 (3) 9.32 (0.92) 4.30 8.68 18.37 33.52

MCPP 107 (97) 3 (3) 1.58 (0.12) 0.91 1.47 2.63 4.30

MECPP 107 (97) 3 (3) 15.91 (1.18) 9.92 14.69 21.77 38.93

MEHHP 107 (97) 3 (3) 7.13 (0.65) 4.15 6.62 11.93 20.99

MEHP 107 (97) 3 (3) 2.60 (0.39) 1.24 2.69 5.58 12.50

MEOHP 107 (97) 3 (3) 4.48 (0.39) 2.59 4.12 7.25 13.27

MEP2 107 (97) 3 (3) 13.17 (1.13) 7.19 11.74 21.09 37.83

MHBP 107 (97) 3 (3) 0.80 (0.11) 0.46 0.99 1.72 2.71

MHIBP 107 (97) 3 (3) 2.91 (0.25) 1.60 2.89 4.94 7.99

MHINCH 107 (97) 3 (3) 1.41 (0.15) 0.65 1.34 2.55 5.71

MIBP 107 (97) 3 (3) 6.22 (0.56) 3.37 6.27 11.83 18.68

MNP2 107 (97) 3 (3) 0.58 (0.07) 0.33 0.53 1.16 2.58

***Others***

DEA 85 (77) 12 (11) 13 (12) 1.35 (0.16) 0.56 1.28 2.30 7.09

Lead 70 (64) 20 (18) 20 (18) 0.66 (0.07) 0.45 0.72 1.10 1.70
