# Supplementary material for: Childhood exposures to environmental chemicals and neurodevelopmental outcomes in congenital heart disease
Source: PLoS One. 2022 Nov 17;17(11):e0277611. doi: 10.1371/journal.pone.0277611 (PMC9671412; doi:10.1371/journal.pone.0277611)
Supplement: S2 Table — (DOCX) [file pone.0277611.s003.docx]

**S2 Table: Intercorrelation Matrix of Analytes (*N* = 110)** Draft Date: 27 Sep 22v2

CPM OPM DEA 24DCP 25DCP

Analyte *n* *r* (*p*-value) *n* *r* (*p*-value) *n* *r* (*p*-value) *n* *r* (*p*-value) *n* *r* (*p*-value)

***Phenols, Parabens, Trichlocarbans***

24DCP 104 0.2993 (0.0020) 96 0.3534 (0.0004)

25DCP 107 0.3827 (< 0.0001)

BPA

BPB

PPB

TCC

TCS 107 0.6207 (< 0.0001)

***Phenols, Parabens, Trichlocarbans***

MBP

MBZP2 104 0.3174 (0.0010)

MCNP

MCOCH

MCOP 107 0.3251 (0.0006)

MCPP 107 0.3699 (< 0.0001)

MECPP 104 0.3740 (< 0.0001)

MEHHP 104 0.4238 (< 0.0001)

MEHP

MEOHP 104 0.4522 (< 0.0001)

MEP2 107 0.3032 (0.0015)

MHBP 104 0.3213 (0.0009) 96 0.3554 (0.0004) 107 0.3527 (0.0002) 107 0.4176 (< 0.0001)

MHIBP 104 0.3789 (< 0.0001) 107 0.3626 (0.0001)

MHINCH

MIBP 104 0.4015 (< 0.0001)

MNP2

BPA BPB PPB TCS MBP

Analyte *n* *r* (*p*-value) *n* *r* (*p*-value) *n* *r* (*p*-value) *n* *r* (*p*-value) *n* *r* (*p*-value)

***Phenols, Parabens, Trichlocarbans***

24DCP

25DCP

BPA

BPB

PPB

TCC 107 0.3221 (0.0007)

TCS 107 0.4873 (< 0.0001) 107 0.2933 (0.0022) 107 0.2925 (0.0022)

***Phenols, Parabens, Trichlocarbans***

MBP

MBZP2 107 0.4286 (< 0.0001)

MCNP 107 0.3678 (< 0.0001)

MCOCH

MCOP 107 0.3524 (0.0002)

MCPP

MECPP

MEHHP 107 0.2967 (0.0019)

MEHP 107 0.5056 (< 0.0001)

MEOHP 107 0.3079 (0.0013)

MEP2 107 0.3025 (0.0015)

MHBP 107 0.5306 (< 0.0001)

MHIBP 107 0.3446 (0.0003)

MHINCH

MIBP 107 0.4018 (< 0.0001)

MNP2

MBZP2 MCNP MCOCH MCOP MCPP

Analyte *n* *r* (*p*-value) *n* *r* (*p*-value) *n* *r* (*p*-value) *n* *r* (*p*-value) *n* *r* (*p*-value)

***Phenols, Parabens, Trichlocarbans***

24DCP

25DCP

BPA

BPB

PPB

TCC

TCS

***Phenols, Parabens, Trichlocarbans***

MBP

MBZP2

MCNP 107 0.3475 (0.0002)

MCOCH

MCOP 107 0.3502 (0.0002) 107 0.7249 (< 0.0001)

MCPP 107 0.4762 (< 0.0001) 107 0.6093 (< 0.001) 107 0.7499 (< 0.0001)

MECPP 107 0.5515 (< 0.0001) 107 0.4784 (< 0.0001) 107 0.4016 (< 0.0001) 107 0.5269 (< 0.0001)

MEHHP 107 0.6566 (< 0.0001) 107 0.4315 (< 0.0001) 107 0.3802 (< 0.0001) 107 0.5741 (< 0.0001)

MEHP

MEOHP 107 0.6569 (< 0.0001) 107 0.4069 (< 0.0001) 107 0.3718 (< 0.0001) 107 0.5507 (< 0.0001)

MEP2 107 0.4280 (< 0.0001) 107 0.3772 (< 0.0001) 107 0.3277 (0.0006) 107 0.4274 (0.0001)

MHBP 107 0.5470 (< 0.0001) 107 0.4392 (< 0.0001)

MHIBP 107 0.4890 (< 0.0001) 107 0.4471 (< 0.0001)

MHINCH 107 0.8902 (< 0.0001)

MIBP 107 0.5276 (< 0.0001) 107 0.4342 (< 0.0001)

MNP2 107 0.3029 (0.0015) 107 0.3302 (0.0005) 107 0.3318 (0.0005)

MECPP MEHHP MEHP MEOHP MEP2

Analyte *n* *r* (*p*-value) *n* *r* (*p*-value) *n* *r* (*p*-value) *n* *r* (*p*-value) *n* *r* (*p*-value)

***Phenols, Parabens, Trichlocarbans***

24DCP

25DCP

BPA

BPB

PPB

TCC

TCS

***Phenols, Parabens, Trichlocarbans***

MBP

MBZP2

MCNP

MCOCH

MCOP

MCPP

MECPP

MEHHP 107 0.8527 (< 0.0001)

MEHP

MEOHP 107 0.8683 (< 0.0001) 107 0.9762 (< 0.0001)

MEP2 107 0.3831 (< 0.0001) 107 0.4631 (< 0.0001) 107 0.4417 (< 0.0001)

MHBP 107 0.4354 (< 0.0001) 107 0.6206 (< 0.0001) 107 0.6288 (< 0.0001) 107 0.4572 (< 0.0001)

MHIBP 107 0.3934 (< 0.0001) 107 0.5470 (< 0.0001) 107 0.5679 (< 0.0001) 107 0.4665 (< 0.0001)

MHINCH

MIBP 107 0.4327 (< 0.0001) 107 0.6000 (< 0.0001) 107 0.6190 (< 0.0001) 107 0.5370 (< 0.0001)

MNP2 107 0.5851 (< 0.0001)

MHBP MHIBP

Analyte *n* *r* (*p*-value) *n* *r* (*p*-value)

***Phenols, Parabens, Trichlocarbans***

24DCP

25DCP

BPA

BPB

PPB

TCC

TCS

***Phenols, Parabens, Trichlocarbans***

MBP

MBZP2

MCNP

MCOCH

MCOP

MCPP

MECPP

MEHHP

MEHP

MEOHP

MEP2

MHBP

MHIBP 107 0.7375 (< 0.0001)

MHINCH

MIBP 107 0.7291 (< 0.0001) 107 0.9164 (< 0.0001)

MNP2
