# Supplementary material for: Childhood exposures to environmental chemicals and neurodevelopmental outcomes in congenital heart disease
Source: PLoS One. 2022 Nov 17;17(11):e0277611. doi: 10.1371/journal.pone.0277611 (PMC9671412; doi:10.1371/journal.pone.0277611)
Supplement: S3 Table — (DOCX) [file pone.0277611.s004.docx]

| **S3 Table: Comparison to Existing Population Exposure Data** | | | | |
| --- | --- | --- | --- | --- |
| **Study** |  | **Canadian Early Life Exposures(3)** | **Switzerland Infant Exposures (4)** | **CHOP Environmental Exposures** |
| **Age at Assessment** |  | ***12 months of age*** | ***6-36 months of age*** | ***18 months of age*** |
| **Years of Assessment** |  | ***2008 - 2012*** | ***2019*** | ***2011 - 2015*** |
| **Units** |  | ***Geometric Mean (SD) ng/ml*** | ***Geometric Mean* μ*g/L*** | ***Geometric Mean (95% CL) μg/L*** |
| **Analytes** | MEP | 12.4 (3.3) | 36.1 (3.7) | 13.2 (11.1, 15.6) |
|  | MBP | 24.3 (2.2) | 54.9 (3.0) | 17.1 (14.6,20.04) |
|  | MBzP | 5.6 (3.7) |  | 4.3 (3.51, 5.28) |
|  | MEHP | 1.8 (2.4) |  | 2.6 (1.98, 3.51) |
|  | MEOHP | 5.5 (3.3) |  | 4.47 (3.77, 5.34) |
|  | MEHHP | 7.8 (2.4) |  | 7.13 (5.95, 8.54) |
|  | MCPP | 1.9 (2.2) | 7.9 (2.5) | 1.58 (13.7, 18.4) |
