# Supplementary material for: Childhood exposures to environmental chemicals and neurodevelopmental outcomes in congenital heart disease
Source: PLoS One. 2022 Nov 17;17(11):e0277611. doi: 10.1371/journal.pone.0277611 (PMC9671412; doi:10.1371/journal.pone.0277611)
Supplement: S4 Table — (DOCX) [file pone.0277611.s005.docx]

| S4 Table: Cohort Characteristics (overall and stratified by cluster) | | | | | | | | |
| --- | --- | --- | --- | --- | --- | --- | --- | --- |
|  | **Overall** | **Cluster 1** | **Cluster 2** | **Cluster 3** | **Cluster 4** | **Cluster 5** | **Cluster 6** | **p-value** |
|  | 100% (107) | 27.10% (29) | 26.17% (28) | 3.73% (4) | 6.54% (7) | 19.62% (21) | 16.82% (18) |  |
| Sex |  |  |  |  |  |  |  | 0.97 |
| Female | 37.38% (40) | 37.93% (11) | 42.86% (12) | 25.00% (1) | 28.57% (2) | 33.33% (7) | 38.89% (7) |  |
| Male | 62.61% (67) | 62.07% (18) | 57.14% (16) | 75.00% (3) | 71.43% (5) | 66.67% (14) | 61.11% (11) |  |
| Race |  |  |  |  |  |  |  | 0.6 |
| Black | 5.61% (6) | 6.90% (2) | 0.00% (0) | 0.00% (0) | 14.29% (1) | 4.76% (1) | 11.11% (2) |  |
| Other | 14.02% (15) | 17.24% (5) | 7.14% (2) | 0.00% (0) | 14.29% (1) | 19.05% (4) | 16.67% (3) |  |
| White | 80.37% (86) | 75.86% (22) | 92.86% (26) | 100.00% (4) | 71.43% (5) | 76.19% (16) | 72.22% (13) |  |
| Ethnicity |  |  |  |  |  |  |  | 0.64 |
| Hispanic | 14.95% (16) | 17.24% (5) | 10.71% (3) | 0.00% (0) | 28.57% (2) | 9.52% (2) | 22.22% (4) |  |
| Non-Hispanic | 85.04% (91) | 82.76% (24) | 89.29% (25) | 100.00% (4) | 71.43% (5) | 90.48% (19) | 77.78% (14) |  |
| Education |  |  |  |  |  |  |  | 0.33 |
| <High School | 5.61% (6) | 6.90% (2) | 3.57% (1) | 25.00% (1) | 14.29% (1) | 4.76% (1) | 0.00% (0) |  |
| Degree/Graduate School | 70.01 (75) | 65.52% (19) | 71.43% (20) | 50.00% (2) | 71.43% (5) | 85.71% (18) | 61.11% (11) |  |
| High School/Some College | 24.30% (26) | 27.59% (8) | 25.00% (7) | 25.00% (1) | 14.29% (1) | 9.52% (2) | 38.89% (7) |  |
| Smoking Status |  |  |  |  |  |  |  | 0.12 |
| Non-smoker | 90.65% (97) | 86.21% (25) | 92.86% (26) | 75.00% (3) | 71.43% (5) | 100.00% (21) | 94.44% (17) |  |
| Smoker | 9.35% (10) | 13.79% (4) | 7.14% (2) | 25.00% (1) | 28.57% (2) | 0.00% (0) | 5.56% (1) |  |
| Hollingshead Score, mean (sd) | 46.22 (13.20) | 42.36 (14.31) | 48.95 (11.26) | 49.50 (17.14) | 41.71 (15.56) | 47.93 (11.47) | 47.19 (14.23) | 0.4 |
