# Supplementary material for: Childhood exposures to environmental chemicals and neurodevelopmental outcomes in congenital heart disease
Source: PLoS One. 2022 Nov 17;17(11):e0277611. doi: 10.1371/journal.pone.0277611 (PMC9671412; doi:10.1371/journal.pone.0277611)
Supplement: S1 Fig — (DOCX) [file pone.0277611.s006.docx]

**Figures**

**Supplemental Figure 1**

**Heading:** The posterior distribution of proportion of individuals falling into a tertile of exposure for each chemical, within each of the six clusters that were found.

**
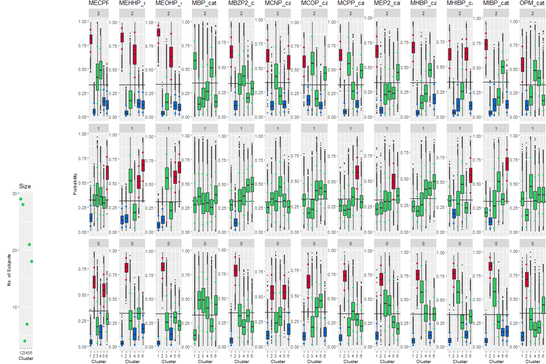
**
